# Takayasu Arteritis in a G6PD Deficient Female Patient With Recurrent Axillary Artery Stenosis: A Case Report

**DOI:** 10.1002/ccr3.73403

**Published:** 2026-09-09

**Authors:** Junaid Imran, Mohammad Zohaib Rehman, Muhammad Osama, Safeena Khan, Pawan Kumar Thada

**Affiliations:** ^1^ Hayatabad Medical Complex Peshawar Pakistan; ^2^ Khyber Medical College Peshawar Pakistan; ^3^ Sotang Primary Hospital Solukhumbu Nepal

**Keywords:** arterial restenosis, axillary artery stenosis, case report, G6PD deficiency, glucose‐6‐phosphate dehydrogenase deficiency, large‐vessel vasculitis, Takayasu arteritis

## Abstract

Takayasu arteritis should be suspected in young females with limb claudication, absent pulses, recurrent arterial stenosis, and elevated inflammatory markers. Early diagnosis is crucial to prevent complications. In G6PD‐deficient females, treatment must be modified, as specific immunosuppressants, like cyclophosphamide, can induce hemolysis. Accurate diagnosis ensures effective management.

## 1 Introduction

Takayasu arteritis (TA) is a rare autoimmune vasculitis that primarily affects the aorta and its branches, with a predilection for young Asian women under 40 years old [1]. Often referred to as the “pulseless disease,” TA is characterized by diminished pulses in the upper extremities, posing diagnostic challenges for healthcare professionals [2]. Despite a prevalence of 2.6 cases per million in the United States, higher rates are reported in Asian countries, Mexico, and Japan [3].

Takayasu arteritis, if left untreated, can lead to severe complications. Therefore, early diagnosis and prompt treatment are crucial for managing patients with TA [3]. TA is primarily treated with corticosteroids and immunosuppressants, such as methotrexate and cyclophosphamide. If the disease progresses to the fibrotic phase causing severe luminal narrowing, revascularization becomes the mainstay of treatment [4]. Depending on the anatomy and extent of the lesion, this can be achieved via minimally invasive endovascular interventional techniques (such as balloon angioplasty and stenting) or traditional open surgical approaches (such as autologous bypass grafting from non‐aortic branch arteries). These methods are associated with varying degrees of success [5].

This case report focuses on an unusual manifestation of TA in a G6PD‐deficient female patient with recurrent axillary artery stenosis. Such unique presentations underscore the importance of increased awareness among clinicians to facilitate early diagnosis and management. Reporting this case contributes to the limited literature on the intersection of TA, G6PD deficiency, and recurrent artery stenosis, offering insights that may enhance clinical understanding and patient care.

## 2 Case Presentation

### 2.1 Case History

A 25‐year‐old female was referred to the medicine department due to an absent pulse and unrecordable blood pressure in her left upper limb. She had initially presented to the gynecology department with severe right iliac fossa pain, nausea, and vomiting; pregnancy‐related causes were ruled out, and an abdominal ultrasound attributed the pain to a non‐surgical hemorrhagic ovarian cyst, which was managed conservatively.

In the medicine department, the patient complained of numbness and claudication in her left upper limb, along with fatigue, weight loss, and bluish discoloration, exacerbated by exertion and cold weather. Her past medical history was notable for G6PD deficiency, diagnosed 5 years earlier. Other history was non‐contributory.

Twelve years prior, the patient underwent bypass surgery for left axillary artery stenosis using an internal mammary artery graft, but the symptoms never improved. She had been on aspirin and warfarin until G6PD deficiency was diagnosed 5 years ago. Family history included rheumatoid arthritis.

### 2.2 Examination

On examination, radial pulses were absent bilaterally, with unrecordable blood pressure in the left arm and 90/60 mmHg in the right arm. Notably, despite the absent right radial pulse, the right upper limb was completely asymptomatic, showing normal skin temperature, normal capillary refill time, and no active claudication or paresthesia, indicating adequate collateral arterial perfusion on the right side. Supraclavicular and shoulder joint tenderness was noted on the left side, along with multiple bruises on the forearms. Auscultation revealed normal heart sounds without murmurs or bruits.

Other commonly involved vascular beds, including the carotid, renal, and mesenteric arteries, were also normal, with no evidence of stenosis, bruits, or ischemic signs, indicating that the disease process was confined to the upper extremities. Additionally, there were no signs of ischemia or claudication in the lower limbs, with normal pulses palpable.

### 2.3 Investigations

**FIGURE 1:**
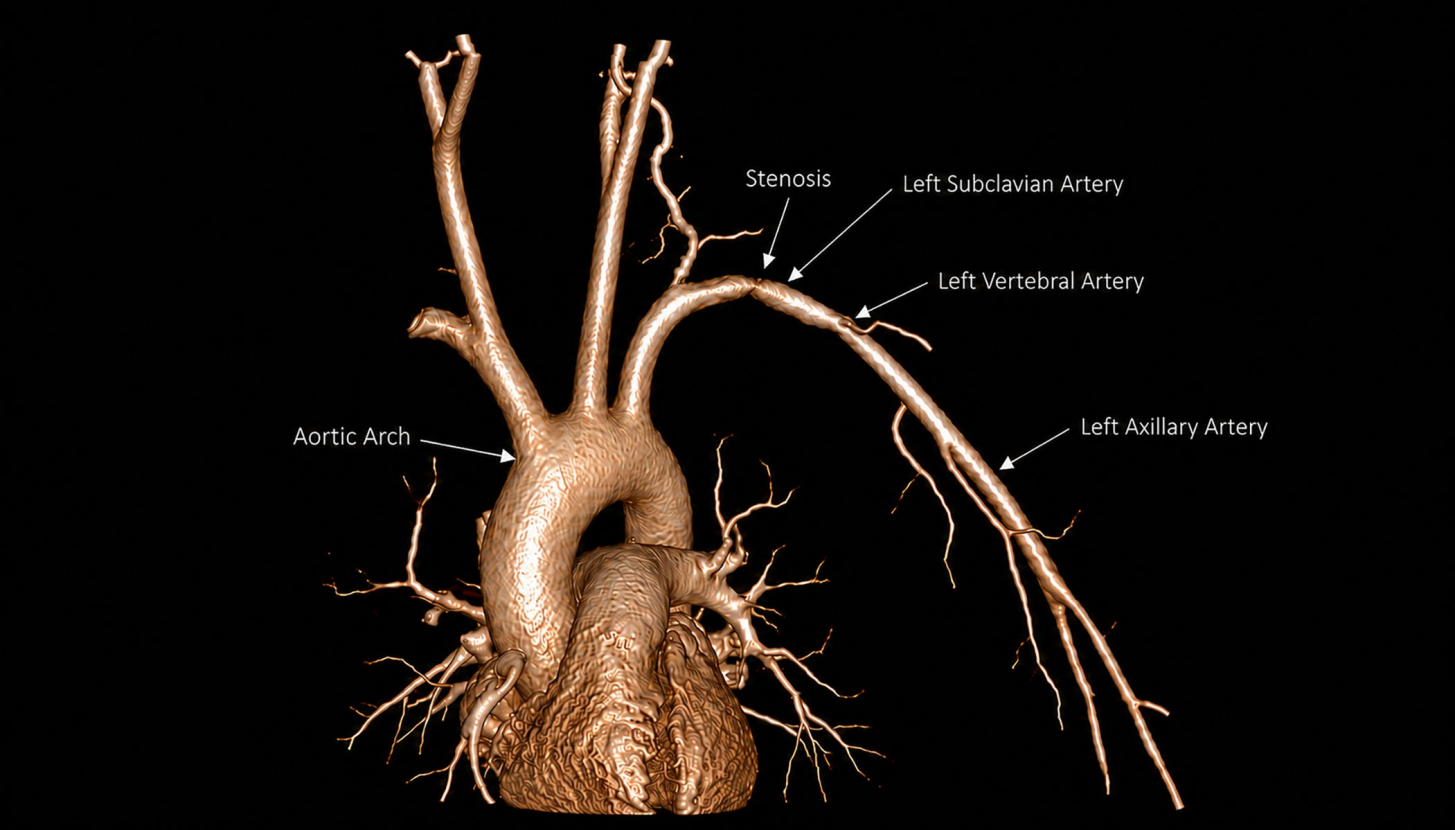
(3D‐CT Angiogram) Thrombosed graft with stenosis in the distal left subclavian artery.

Laboratory investigations revealed a hemoglobin level of 9.5 g/dL, an elevated ESR (144 mm/h), and positive Anti‐smooth muscle antibodies (ASMA) and Anti‐Golgi apparatus antibodies (Tables 1 and 2). Imaging studies, including Doppler ultrasound and CT angiogram (Figure 1), confirmed a thrombosed graft and narrowing in the left axillary and subclavian arteries.

**TABLE 1:** Baseline investigations of the patient.

Investigation	Result	Normal range
TLC	9.2	4–11
DLC	Neutrophils: 81%	40%–75%
	Lymphocytes: 15%	20%–45%
	Monocytes: 2%	2%–10%
Hemoglobin	9.6 g/dL	12–16 g/dL
Platelets	264 × 10^3^/μL	150–450 × 10^3^/μL
Coagulation profile	PT: 16.0 s	Control: 14.0 s
	INR: 1.1	Control: 1
	APTT: 32.0 s	Control: 30.0 s
Blood urea	16.7 mg/dL	10–50 mg/dL
Creatinine	0.59 mg/dL	0.1–1.0 mg/dL
Liver profile	Bilirubin: 0.28 mg/dL	0.1–1.0 mg/dL
	ALT: 9.8 U/L	10–50 U/L
	ALP: 23 U/L	35–104 U/L
ESR	141 mm/1st hr.	0–20 mm/1st hr.
CRP	0.98 mg/L	< 5.0 mg/L
Serum electrolytes	Sodium: 104 mmol/L	135–150 mmol/L
	Potassium: 3.9 mmol/L	3.5–5.0 mmol/L
	Chloride: 104 mmol/L	98–108 mmol/L
D—Dimers	489 ng/mL	< 500 ng/mL
Viral profile	HbsAg: Negative	Negative
	Anti‐HCV: Negative	Negative
	Anti‐HIV: Negative	Negative
B‐HCG (Titer)	1.20 mIU/mL	0.0–10.0 mIU/mL
Vitamin B‐12	190.6 pg/mL	193–982 pg/mL

**TABLE 2:** Autoimmune profile of the patient.

Autoimmune profile		
Antibody	Result	Normal
ANA (antinuclear antibodies)	Negative	Negative
AMA (antimitochondrial antibodies)	Negative	Negative
ASMA (anti‐smooth muscle antibodies)	Positive	Negative
GPC (gastric parietal cell antibodies)	Negative	Negative
LKM (liver kidney microsomal antibody)	Negative	Negative
AGAs (anti‐golgi autoantibodies)	Positive	Negative

Clinical findings, examination, and the updated American College of Rheumatology (ACR) criteria for the diagnosis of Takayasu arteritis (2022) supported the definitive diagnosis of Takayasu arteritis [6].

### 2.4 Treatment and Follow‐Up

The patient was started on corticosteroids and methotrexate. Cyclophosphamide was avoided due to the risk of triggering hemolysis in G6PD deficiency, which could result in severe complications. This medication was excluded as it induces oxidative stress, potentially worsening hemolytic anemia. She was referred to a vascular surgeon, who initially deferred angioplasty and recommended medical management. The patient was regularly followed up with laboratory tests, showing significant clinical improvement by the second visit 4 months later. Notable improvements included reduced claudication, restored pulses, and decreased inflammatory markers, reflecting a positive response to treatment.

### 2.5 Differential Diagnosis

Differential diagnoses considered include giant cell arteritis (unlikely due to age), Buerger's disease (associated with male smokers), antiphospholipid syndrome (absence of antibodies), atherosclerosis (absence of risk factors), systemic lupus erythematosus (negative ANA), and fibromuscular dysplasia (absence of typical imaging findings). Overall, clinical, laboratory, and radiologic evidence strongly support Takayasu arteritis as the primary diagnosis.

## 3 Discussion

Takayasu arteritis (TA) is a chronic inflammatory vasculitis that primarily affects large arteries, particularly the aorta and its branches, including the axillary artery [6]. It is one of the most common types of vasculitis, involving the large arteries of the upper extremities, and can lead to serious ischemic complications, such as claudication, paresthesia, pain, and Raynaud's phenomenon [7]. Recurrent axillary artery stenosis after an arterial graft is uncommon but has been documented in the literature. The exact underlying cause is not known, but it is often multifactorial, including progressive vascular inflammation, neointimal hyperplasia, and recurrent thrombosis [8]. However, in this case, a normal lipid profile ruled out atherosclerosis and a normal coagulation profile excluded a hypercoagulable state as a contributing factor. Given the patient's young age, elevated ESR, family history of autoimmune disease, and characteristic imaging findings, the diagnosis of Takayasu arteritis was strongly supported.

The delayed diagnosis of Takayasu arteritis (TA) in this patient occurred because the initial features of the disease were overlooked, and the signs were mistakenly attributed to arterial stenosis, for which the patient underwent graft surgery. The persistent symptoms, despite surgery, further clouded the true diagnosis. Additionally, the patient's G6PD deficiency prevented the use of certain immunosuppressive medications, such as cyclophosphamide, which could have potentially unmasked the underlying TA. This combination of misinterpretation of symptoms and limited treatment options led to a delay in the correct diagnosis, underscoring the importance of considering TA in patients with recurrent vascular symptoms.

This case highlights the challenges in managing recurrent arterial stenosis in TA, especially when standard interventions such as arterial grafting fail to provide long‐term relief. While bypass grafts and angioplasty can help restore blood flow, they do not address the underlying inflammatory process that drives disease progression [9]. Without adequate immunosuppressive therapy, the inflammation of vessels can persist, leading to restenosis and graft failure. The recurrence of stenosis in our patient, despite previous surgical intervention, reinforces the importance of long‐term medical management alongside vascular procedures.

A particularly important aspect of this case is the coexistence of TA with glucose‐6‐phosphate dehydrogenase (G6PD) deficiency, which does not appear to be pathophysiologically related to TA but significantly influences treatment selection [10]. Managing TA in G6PD‐deficient patients requires careful drug selection and monitoring, as immunosuppressants such as cyclophosphamide carry a risk of triggering hemolysis and can further compromize the patient's health [11]. This makes treatment more challenging since the medications chosen must control the disease without triggering oxidative stress.

Another noteworthy finding in this case was the presence of anti‐smooth muscle antibodies (ASMA) and anti‐Golgi apparatus antibodies, which are more commonly associated with autoimmune liver diseases. Their role in TA is unclear, but their presence suggests a broader autoimmune dysregulation. Whether these antibodies contribute to vascular inflammation or are incidental findings remains uncertain, highlighting the need for further research into the immunological landscape of TA [12, 13].

This case underscores the importance of a multidisciplinary approach in managing Takayasu arteritis, particularly in patients with rare comorbidities that complicate standard treatment protocols. It also highlights the need for ongoing research to understand better the mechanisms underlying graft failure in TA and to develop evidence‐based strategies for long‐term vascular management.

## 4 Conclusion

In conclusion, this case reinforces the importance of considering Takayasu arteritis in young patients presenting with recurrent axillary artery stenosis. Early recognition and appropriate intervention are essential to prevent further vascular complications. While immunosuppressive therapy remains the cornerstone of treatment, surgical interventions may still be necessary for severe stenosis or graft failure. The presence of G6PD deficiency in this patient further complicates treatment decisions, emphasizing the need for individualized therapy and close monitoring. Continued research is required better to understand the interplay between TA, vascular interventions, and coexisting metabolic disorders, ultimately improving patient outcomes.

## Author Contributions

**Junaid Imran:** conceptualization, writing – original draft, writing – review and editing, methodology. **Muhammad Osama:** data curation, conceptualization, writing – original draft, writing – review and editing, methodology. **Pawan Kumar Thada:** methodology, data curation, supervision, writing – review and editing, writing – original draft. **Safeena Khan:** writing – review and editing, writing – original draft, methodology. **Mohammad Zohaib Rehman:** conceptualization, writing – original draft, writing – review and editing, formal analysis.

## Funding

The authors have nothing to report.

## Consent

Written informed consent was taken from the patient.

## Conflicts of Interest

The authors declare no conflicts of interest.

## Data Availability

The data that support the findings of this study are available from the corresponding author upon reasonable request.
